# Myosteatosis is associated with poor physical fitness in patients undergoing hepatopancreatobiliary surgery

**DOI:** 10.1002/jcsm.12433

**Published:** 2019-05-21

**Authors:** Malcolm A. West, David P.J. van Dijk, Fredrick Gleadowe, Thomas Reeves, John N. Primrose, Mohammed Abu Hilal, Mark R. Edwards, Sandy Jack, Sander S.S. Rensen, Michael P.W. Grocott, Denny Z.H. Levett, Steven W.M. Olde Damink

**Affiliations:** ^1^ Academic Unit of Cancer Sciences, Faculty of Medicine University of Southampton Southampton UK; ^2^ Integrative Physiology and Critical Illness Group, Clinical and Experimental Sciences, Faculty of Medicine University of Southampton Southampton UK; ^3^ Respiratory and Critical Care Research Theme, Southampton NIHR Biomedical Research Centre University Hospital Southampton NHS Foundation Trust, Anaesthesia and Critical Care Southampton UK; ^4^ Department of Surgery Maastricht University Medical Centre Maastricht The Netherlands; ^5^ NUTRIM School of Nutrition and Translational Research in Metabolism Maastricht University Maastricht The Netherlands; ^6^ Departments of General, Visceral and Transplantation Surgery RWTH University Hospital Aachen Aachen Germany

**Keywords:** Body composition, Cardiopulmonary exercise testing, Physical fitness, Sarcopenia, Myosteatosis, Oxygen uptake

## Abstract

**Background:**

Body composition assessment, measured using single‐slice computed tomography (CT) image at L3 level, and aerobic physical fitness, objectively measured using cardiopulmonary exercise testing (CPET), are each independently used for perioperative risk assessment. Sarcopenia (i.e. low skeletal muscle mass), myosteatosis [i.e. low skeletal muscle radiation attenuation (SM‐RA)], and impaired objectively measured aerobic fitness (reduced oxygen uptake) have been associated with poor post‐operative outcomes and survival in various cancer types. However, the association between CT body composition and physical fitness has not been explored. In this study, we assessed the association of CT body composition with selected CPET variables in patients undergoing hepatobiliary and pancreatic surgery.

**Methods:**

A pragmatic prospective cohort of 123 patients undergoing hepatobiliary and pancreatic surgery were recruited. All patients underwent preoperative CPET. Preoperative CT scans were analysed using a single‐slice CT image at L3 level to assess skeletal muscle mass, adipose tissue mass, and muscle radiation attenuation. Multivariate linear regression was used to test the association between CPET variables and body composition. Main outcomes were oxygen uptake at anaerobic threshold ( $\dot{V}$O_2_ at AT), oxygen uptake at peak exercise ( $\dot{V}$O_2_ peak), skeletal muscle mass, and SM‐RA.

**Results:**

Of 123 patients recruited [77 men (63%), median age 66.9 ± 11.7, median body mass index 27.3 ± 5.2], 113 patients had good‐quality abdominal CT scans available and were included. Of the CT body composition variables, SM‐RA had the strongest correlation with $\dot{V}$O_2_ peak (*r* = 0.57, *P* < 0.001) and $\dot{V}$O_2_ at AT (*r* = 0.45, *P* < 0.001) while skeletal muscle mass was only weakly associated with $\dot{V}$O_2_ peak (*r* = 0.24, *P* < 0.010). In the multivariate analysis, only SM‐RA was associated with $\dot{V}$O_2_ peak (*B* = 0.25, 95% CI 0.15–0.34, *P* < 0.001, *R* ^2^ = 0.42) and $\dot{V}$O_2_ at AT (*B* = 0.13, 95% CI 0.06–0.18, *P* < 0.001, *R* ^2^ = 0.26).

**Conclusions:**

There is a positive association between preoperative CT SM‐RA and preoperative physical fitness ( $\dot{V}$O_2_ at AT and at peak). This study demonstrates that myosteatosis, and not sarcopenia, is associated with reduced aerobic physical fitness. Combining both myosteatosis and physical fitness variables may provide additive risk stratification accuracy and guide interventions during the perioperative period.

## Background

Despite improvement in surgical techniques, multimodal cancer therapies and perioperative care, morbidity, and mortality after major hepatobiliary and pancreatic (HPB) cancer surgery still pose substantial challenges. Accurate perioperative risk assessment prior to major cancer surgery is not personalized and is still substantially variable in the precision of outcome prediction. Identifying patients who are at risk of poor outcomes is a priority in order to facilitate shared decision making, inform prehabilitation and co‐morbidity management initiatives before surgery, and guide intra‐operative and post‐operative care. Various scoring systems exist for the risk stratification of these patients; however, few are objective. The importance of objectively measured aerobic physical fitness [using cardiopulmonary exercise testing (CPET)]^1^ and objectively defined body composition variables such as sarcopenia^2^, ^3^, ^4^, ^5^ or myosteatosis^6^ [using routinely performed abdominal computed tomography (CT) scanning] as major contributors to poor post‐operative outcomes and death has so far been studied separately.

CPET provides an objective assessment of physical fitness through evaluating cardio‐respiratory function under the stress of exercise mimicking the stress of major surgery. This has been widely adopted in the UK as a preoperative test to objectively evaluate perioperative risk,^7^ and international guidelines for CPET conduct have recently been published.^8^ The ability of CPET to identify patients at risk of poor outcomes is used clinically to guide perioperative care and clinical decision making, and it informs about the shared decision‐making process.^1^, ^9^ We have previously reported that selected CPET variables such as oxygen uptake at estimated lactate threshold or anaerobic threshold ( $\dot{V}$O_2_ at AT) and at peak exercise ( $\dot{V}$O_2_ peak) are associated with worse outcome following colorectal surgery^10^, ^11^, ^12^ and neoadjuvant cancer treatments.^13^, ^14^ Poor physical fitness is highly prevalent in HPB cancer patients and associated with poor post‐operative outcomes and survival.^15^, ^16^, ^17^, ^18^, ^19^

Body composition analysed by using a single‐slice CT image at the third lumbar vertebra (L3) is strongly correlated with total body skeletal muscle mass.^20^ The area of visceral adipose tissue (VAT) and subcutaneous adipose tissue (SAT) can also be accurately estimated using this methodology. In addition, CT scans contain information about the radio density of a specific tissue type in Hounsfield unit (HU), which is referred to as radiation attenuation (RA). Low muscle RA is considered a surrogate of increased intramyocellular triglycerides, increased water content (i.e. muscle oedema), change in muscle structure, and dysregulated host systemic inflammatory response.^21^, ^22^, ^23^ A recent review showed that skeletal muscle RA, referred to as myosteatosis (SM‐RA), is highly prevalent in cancer patients.^23^ Sarcopenia and myosteatosis have been found to be independent prognostic factors of reduced survival and poor outcome after surgery or neoadjuvant treatments in various cancers including pancreatic,^4^, ^24^ colorectal,^25^, ^26^ gastric,^27^ esophageal,^28^, ^29^ and ovarian cancers.^30^ This relationship was less evident in colorectal liver metastasis patients.^31^, ^32^, ^33^, ^34^ It is hypothesized that changes in muscle tissue composition such as low SM‐RA (myosteatosis) results in diminished muscle function phenotypically expressed as poor resilience that may potentially be reversed by improving activity levels or exercise interventions.^5^, ^35^ Therefore, low SM‐RA is often reported as an indicator of ‘poor muscle quality’. However, the association between low SM‐RA and physical fitness has not previously been evaluated. We therefore aimed to assess the association of low SM‐RA (i.e. myosteatosis), low skeletal muscle mass (i.e. sarcopenia), and selected CPET variables (i.e. aerobic physical fitness) in a representative HPB population.

## Methods

### Subjects and data collection

All consecutive patients undergoing CPET and HPB surgery between January 2014 and January 2018, at the University Hospital Southampton NHS Foundation Trust HPB Unit, UK, were included in a prospective cohort and were eligible for inclusion. The study was reviewed and approved by the South East Scotland Research Ethics Committee (16/SS/0188) and is registered with clinicaltrials.gov (NCT03641118). All patients had a histological or radiological diagnosis of operable liver metastases (melanoma, colorectal, breast), periampullary carcinoma, hepatocellular carcinoma, or benign disease necessitating major liver or pancreas resections. A minority of patients underwent neoadjuvant cycles of capecitabine and oxaliplatin prior to colorectal liver metastasis surgery. This represented a pragmatic prospectively collected patient cohort reflecting a busy tertiary HPB referral centre. All patients underwent CPET before surgery. Body composition was assessed using a preoperative single‐slice CT image at the level of the third lumbar vertebra (L3). Patients without a good‐quality preoperative abdominal CT scan were excluded. CT scans were defined as poor quality if they had large radiation artefacts or profound muscle oedema. Preoperative plasma levels of haemoglobin, creatinine, albumin, and C‐reactive protein were assessed within 7 days of the planned surgery. The albumin and C‐reactive protein levels were used to calculate the modified Glasgow prognostic score (mGPS).^36^ Additional data collection included sex, age, body mass index (BMI), American Society of Anesthesiologists (ASA) classification, type of surgery, and histopathology diagnosis. Primary outcomes were $\dot{V}$O_2_ at AT and $\dot{V}$O_2_ peak (mL·kg^−1^·min^−1^). Secondary outcomes included 1 year all‐cause mortality and length of hospital stay; both were measured from day of surgery.

### Cardiopulmonary exercise testing

CPET was conducted according to standardized methods published elsewhere by the Perioperative Exercise Testing and Training Society and endorsed by the Association for Respiratory Technology & Physiology in the UK.^8^ In short, after resting spirometry (flow‐volume loops), CPET on an electromagnetically braked cycle ergometer (Ergoselect 200; Ergoline, Bitz, Germany) comprised 3 min resting (to allow gas exchange variables to stabilize), 3 min freewheel pedalling, and then a ramped incremental protocol until volitional termination followed by 5 min recovery data collection. Ventilation and gas exchange were measured using a metabolic cart. Heart rate, full disclosure 12‐lead electrocardiogram, blood pressure, and pulse oximetry were monitored throughout. Ramp gradient was based on a calculation using predicted freewheel $\dot{V}$O_2_, predicted $\dot{V}$O_2_ peak, height, and age with the aim of achieving a 10 min ramp stage. All CPETs were analysed by experienced accredited perioperative CPET practitioners (D. Z. H. L./M. R. E.) who were blinded to CT scan variables and clinical outcome variables.

### Computed tomography scan analysis

The preoperative CT scan performed nearest to the date of surgery was selected for analysis (max 4 weeks before surgery and after completion of neoadjuvant chemotherapy). Abdominal CT scans were analysed in an anonymized and blinded format by one researcher trained in body composition analysis (D. P. J. V. D.) as described before.^24^ Briefly, a single‐slice CT image at the level of the third lumbar vertebra (L3) was selected. CT scans were assessed with sliceOmatic 5.0 (TomoVision, Magog, Canada) for Microsoft Windows®. With the use of predefined HU ranges, the cross‐sectional areas (cm^2^) of skeletal muscle (SM; −29 to 150 HU), VAT (−150 to −50 HU), and SAT (−190 to −30 HU) were assessed. The cross‐sectional area was then adjusted for height squared to calculate the L3 index (cm^2^·m^−2^), which is strongly correlated with total body SM and adipose tissue mass.^20^ In addition, the average RA in HU was assessed for all tissues (SM‐RA, VAT‐RA, and SAT‐RA). A low RA is associated with increased tissue triglyceride content.^23^ Analyses were blinded to CPET and outcome variables.

### Statistical analysis

Data were analysed using IBM SPSS Statistics 23 for Microsoft Windows. Continuous data were compared using an independent *t*‐test, the Mann–Whitney *U*‐test was used for non‐parametric variables, and the *χ* ^2^ test was used for categorical variables. As the cohort size was too small for cut‐point finding approaches such as optimum stratification, gender‐specific cut points were set at the median for each CT body composition and CPET variable.^37^ The association of body composition and other clinical variables on CPET derived $\dot{V}$O_2_ at AT and $\dot{V}$O_2_ peak was assessed using linear regression. First, all clinical variables were tested separately in an unadjusted univariate model (Model 1). Then, variables were ordered from lowest to highest *P*‐value from Model 1 and were added one by one to the multivariate linear regression model (Model 2). After each addition, an *F*‐test was performed to test whether the added variables significantly improved the model fit. If the *F*‐test *P*‐value was <0.05, the variable was kept in the model; otherwise, it was removed. Univariate and multivariate Cox regression and logistic regression were used for, respectively, overall survival and 1 year survival. Variables with a *P* < 0.1 in the univariate analysis were included into the multivariate analysis. For correlations, Pearson's correlation coefficient was used (*r*) for normally distributed data and the Spearman correlation coefficient (*r*_s_) for non‐normally distributed data.

## Results

### Patients

One hundred twenty‐three patients were included during the study. Liver resections consisted of liver metastases (39 patients; 1 benign and 15 neoadjuvant chemotherapy), hepatocellular carcinoma (5 patients), and other major liver resections (median 2 liver segments resected; 15 patients; 9 patients benign). Pancreatic resections consisted of pancreatic neoplasms (29 patients; 5 benign), ampullary carcinoma (16 patients), cholangiocarcinoma (16 patients; 3 benign), and intraductal papillary mucinous neoplasm (3 patients). Ten patients were excluded for CT scan analysis because they did not have a suitable preoperative CT scan available. Patient characteristics are shown in *Table* 1, including the distribution among skeletal muscle mass, SM‐RA, and the main CPET parameters split into high and low groups at the gender‐specific medians for men and women, respectively: SM mass, 50.7 and 38.4 cm^2^·m^−2^; SM‐RA, 37.1 and 30.4 HU; $\dot{V}$O_2_ at AT, 9.4 and 9.3 mL·kg^−1^·min^−1^; and $\dot{V}$O_2_ peak, 16.0 and 14.3 mL·kg^−1^·min^−1^. Patients with low skeletal muscle mass, SM‐RA, low $\dot{V}$O_2_ at AT, and low $\dot{V}$ O_2_ peak were significantly older. No significant differences could be detected in body composition or fitness variables when comparing benign vs. malignant or liver vs. pancreas groups. Significantly lower haemoglobin was seen in the low $\dot{V}$O_2_ at AT and $\dot{V}$O_2_ peak groups; however, no difference was found in skeletal muscle mass and SM‐RA. No significant differences could be detected in C‐reactive protein, GPS, and white cell counts. However, there were weak correlations between SM‐RA and both acute phase proteins C‐reactive protein (*r*_s_ = −0.22, *P* = 0.02) and albumin (*r*_s_ *=* 0.19, *P* = 0.04).

**Table 1:** Patient characteristics according to several computed tomography body composition and cardiopulmonary exercise testing variables

Patient characteristics	Overall	SM‐RA			$\dot{V}$O_2_ at AT (mL·kg^−1^·min^−1^)			$\dot{V}$O_2_ peak (mL·kg^−1^·min^−1^)		
		Low	High	P‐value	Low	High	P‐value	Low	High	P‐value
Age (years)	66.9 ± 11.7	71.1 ± 8.5	63.0 ± 13.4	<0.001*	70.2 ± 10.1	63.6 ± 12.3	0.002*	71.4 ± 8.8	62.3 ± 12.5	<0.001*
Male (*n*, %)	77 (63%)	36 (63%)	35 (63%)	0.942	39 (63%)	38 (62%)	0.944	39 (63%)	38 (62%)	0.944
BMI (kg·m^−2^)	27.3 ± 5.2	29.1 ± 4.6	26.1 ± 4.3	0.001*	28.1 ± 5.9	26.5 (4.3)	0.082	28.1 ± 5.8	26.5 ± 4.4	0.097
ASA^a^										
2	80 (65%)	36 (63%)	37 (66%)	0.699	36 (58%)	44 (72%)	0.093	34 (55%)	46 (75%)	0.015*
3	42 (34%)	20 (35%)	19 (34%)		25 (40%)	17 (28%)		27 (44%)	15 (25%)	
4	1 (1%)	1 (2%)	—		1 (2%)	—		1 (2%)	—	
Organ (*n*, %)										
Liver	59 (48%)	28 (49%)	29 (52%)	0.777	31 (50%)	28 (46%)	0.649	32 (52%)	27 (44%)	0.415
Pancreas	64 (52%)	29 (51%)	27 (48%)		31 (50%)	33 (54%)		30 (48%)	34 (56%)	
Malignant (*n*, %)	103 (84%)	49 (86%)	47 (84%)	0.762	52 (84%)	51 (84%)	0.968	50 (81%)	53 (87%)	0.348
Haemoglobin (g·L^−1^)	127.8 ± 17.7	128.0 ± 17.0	128.2 ± 19.6	0.963	124.1 ± 17.8	131.5 ± 17.0	0.019*	123.7 ± 18.1	132.0 ± 16.4	0.009*
Creatinine (μmol·L^−1^)	77.9 ± 22.6	81.1 ± 26.1	74.4 ± 19.2	0.123	81.8 ± 26.0	74.0 ± 17.9	0.057	82.9 ± 26.0	72.9 ± 17.3	0.014*
White blood count (10 × 9 L^−1^)	8.1 ± 5.5	7.7 ± 1.8	8.5 ± 7.9	0.428	9.0 ± 7.3	7.2 ± 2.2	0.068	8.8 ± 7.3	7.4 ± 2.4	0.154
C‐reactive protein (mg·L^−1^)^a^^,^ b	12.1 ± 19.9	13.8 ± 20.4	11.7 ± 20.9	0.073	9.6 ± 12.7	14.6 ± 24.8	0.623	11.7 ± 15.1	12.6 ± 23.9	0.063
Albumin (g·L^−1^)^b^	36.2 ± 4.7	35.7 ± 4.2	36.5 ± 5.2	0.348	36.0 ± 4.7	36.3 ± 4.8	0.790	35.6 ± 4.7	36.8 ± 4.7	0.170
mGPS (*n*, %)^a^^,^ b										0.851
0	79 (67%)	37 (69%)	34 (63%)	0.736	39 (69%)	40 (67%)	0.900	39 (67%)	40 (68%)	
1	16 (14%)	5 (9%)	10 (19%)		7 (12%)	9 (15%)		7 (12%)	9 (15%)	
2	22 (19%)	12 (22%)	10 (19%)		11 (19%)	11 (18%)		12 (21%)	10 (17%)	
Hospital stay (days)^a^	12.3 ± 10.3	12.8 ± 11.3	11.9 ± 10.1	0.822	12.8 ± 10.4	11.8 ± 10.3	0.487	13.9 ± 11.0	10.6 ± 9.3	0.048*
1 year mortality (*n*, %)	18 (14.6%)	11 (19%)	7 (13%)	0.323	11 (18%)	7 (12%)	0.326	13 (21%)	5 (8%)	0.045*

Cut‐offs for low and high groups were set at the gender‐specific median for SM‐RA, $\dot{V}$O_2_ at AT (mL·kg^−1^·min^−1^), and $\dot{V}$O_2_ peak (mL·kg^−1^·min^−1^).

ASA, American Society of Anesthesiologists; BMI, body mass index; mGPS, modified Glasgow prognostic score; SM‐RA, skeletal muscle radiation attenuation; $\dot{V}$O_2_ at AT, oxygen uptake at anaerobic threshold; $\dot{V}$O_2_ peak, oxygen uptake at peak exercise.

a Mann–Whitney *U*‐test.

b Missing data: C‐reactive protein (*n* = 6), albumin (*n* = 1), and mGPS (*n* = 7).

* Significant *P*‐value < 0.05.

**Figure 1:**
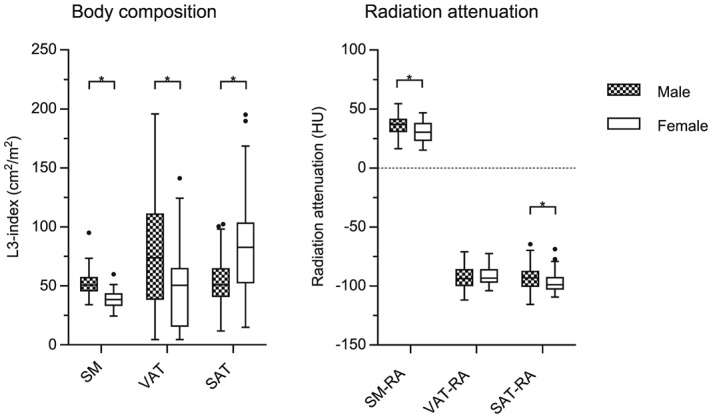
Gender‐specific CT body composition. Boxes represent median and inter‐quartile range. Whiskers are set at either the 25th or 75th percentiles + 1.5 times the inter‐quartile range (Tukey method). Dots represent outliers. *Significant P‐value < 0.05. CT, computed tomography; RA, radiation attenuation; SAT, subcutaneous adipose tissue; SM, skeletal muscle; VAT, visceral adipose tissue.

All 123 patients underwent CPET, of which six patients were not able to achieve an anaerobic threshold (all six died at 1 year follow‐up). No CPET‐related adverse events occurred. For both CT and CPET assessments, there were significant differences between genders. In short, men had higher SM (*P* < 0.001), VAT (*P* < 0.001), SM‐RA (*P* = 0.001), and SAT‐RA (*P* = 0.048), while women had higher SAT (*P* < 0.001); see *Figure* 1. For CPET results, men had higher $\dot{V}$O_2_ at AT (*P* < 0.001), oxygen pulse ( $\dot{V}$O_2_/Heart RateR) at AT (*P* < 0.001), ventilatory equivalent for carbon dioxide at AT ( $\dot{V}$_E_/ $\dot{V}$CO_2_ at AT) (*P* < 0.001), work rate at AT (*P* < 0.001), $\dot{V}$O_2_ peak in litres per minute (*P* < 0.001), $\dot{V}$O_2_ peak adjusted for weight (*P* = 0.033), $\dot{V}$O_2_/Heart Rate at peak (*P* < 0.001), and work rate at peak (*P* < 0.001). *Table* 2 shows gender‐specific comparisons for all CPET variables according to SM‐RA. Weight‐adjusted $\dot{V}$O_2_ at AT for men (*P* = 0.019) but not for women (*P* > 0.05) was significantly higher in the high SM‐RA group. Weight‐adjusted $\dot{V}$O_2_ peak was higher in the high SM‐RA grouped for both men and women (men, *P* = 0.002; women, *P* = 0.008). A higher work rate at AT and peak was also seen in both groups at high SM‐RA values, with significant differences in women (AT *P* = 0.030 and peak *P* = 0.016). Furthermore, a significantly lower $\dot{V}$_E_/ $\dot{V}$CO_2_ slope for both men and women (men, *P* = 0.014 and women, *P* = 0.005) and $\dot{V}$_E_/ $\dot{V}$CO_2_ at AT and peak (*P* = 0.018 and *P* = 0.025) for men were observed in the higher SM‐RA groups.

**Table 2:** Gender‐specific cardiopulmonary exercise testing results according to skeletal muscle radiation attenuation

	Male				Female			
	Overall	Low SM‐RA	High SM‐RA	P‐value	Overall	Low SM‐RA	High SM‐RA	P‐value
$\dot{V}$O_2_ at AT (L·min^−1^)	0.87 ± 0.28	0.82 ± 0.22	0.90 ± 0.34	0.261	0.70 ± 0.19	0.70 ± 0.21	0.70 ± 0.18	0.970
$\dot{V}$O_2_ at AT (mL·kg^−1^·min^−1^)	10.3 ± 3.1	9.4 ± 2.2	11.1 ± 3.6	0.019*	10.0 ± 2.5	9.2 ± 2.0	10.6 ± 2.9	0.100
$\dot{V}$O_2_/Heart Rate at AT (mL per beat)	8.6 ± 2.2	8.7 ± 2.1	8.5 ± 2.4	0.782	6.8 ± 1.7	6.9 ± 1.9	6.7 ± 1.7	0.618
$\dot{V}$_E_/ $\dot{V}$CO_2_ at AT	37.2 ± 6.1	39.0 ± 6.1	35.6 ± 5.7	0.018*	37.2 ± 6.9	39.5 ± 8.1	35.2 ± 5.6	0.067
Work rate at AT (W)	62.9 ± 27.9	56.1 ± 22.3	68.5 ± 31.1	0.060	45.4 ± 23.7	38.5 ± 23.4	54.0 ± 21.3	0.030*
$\dot{V}$O_2_ peak (L·min^−1^)	1.41 ± 0.50	1.29 ± 0.34	1.50 ± 0.60	0.074	1.05 ± 0.28	1.01 ± 0.32	1.09 ± 0.26	0.359
$\dot{V}$O_2_ peak (mL·kg^−1^·min^−1^)	16.9 ± 5.4	14.8 ± 3.6	18.9 ± 6.3	0.002*	15.0 ± 3.6	13.5 ± 2.5	16.4 ± 4.1	0.008*
$\dot{V}$O_2_/Heart Rate at peak (mL per beat)	10.7 ± 2.6	10.6 ± 2.6	10.8 ± 2.8	0.780	8.1 ± 2.2	8.4 ± 2.7	7.9 ± 1.7	0.458
Work rate at peak (W)	126.1 ± 48.7	113.5 ± 36.3	135.9 ± 56.4	0.052	89.4 ± 35.3	75.9 ± 32.6	102.4 ± 35.7	0.016*
$\dot{V}$_E_/ $\dot{V}$ CO_2_ at peak	36.1 ± 5.6	37.6 ± 5.6	34.6 ± 5.4	0.025*	36.1 ± 6.5	38.1 ± 7.4	34.7 ± 5.6	0.104
$\dot{V}$O_2_/work rate slope (mL·min^−1^·W^−1^)	9.4 ± 1.4	9.2 ± 1.5	9.6 ± 1.4	0.206	9.3 ± 1.6	9.5 ± 1.6	9.3 ± 1.7	0.812
$\dot{V}$_E_/ $\dot{V}$CO_2_ slope	32.0 ± 5.6	33.7 ± 5.8	30.4 ± 5.1	0.014*	32.2 ± 6.8	35.1 ± 7.3	29.4 ± 4.7	0.005*

Cut‐offs for low and high groups were set at the gender‐specific median for SM‐RA.

SM‐RA, skeletal muscle radiation attenuation; $\dot{V}$O_2_ at AT, oxygen uptake at anaerobic threshold; $\dot{V}$O_2_ peak, oxygen uptake at peak exercise; $\dot{V}$O_2_/Heart R at AT/peak, oxygen pulse at the anaerobic threshold/peak exercise; $\dot{V}$_E_/ $\dot{V}$CO_2_ at AT/peak, ventilatory equivalent for carbon dioxide at the anaerobic threshold/peak exercise; $\dot{V}$O_2_/work rate slope, oxygen uptake over work rate slope; $\dot{V}$_E_/ $\dot{V}$CO_2_ slope, ventilation over ventilatory equivalent for carbon dioxide.

* Significant *P*‐value < 0.05.

### Relationship between computed tomography body composition and cardiopulmonary exercise testing variables

**Figure 2:**
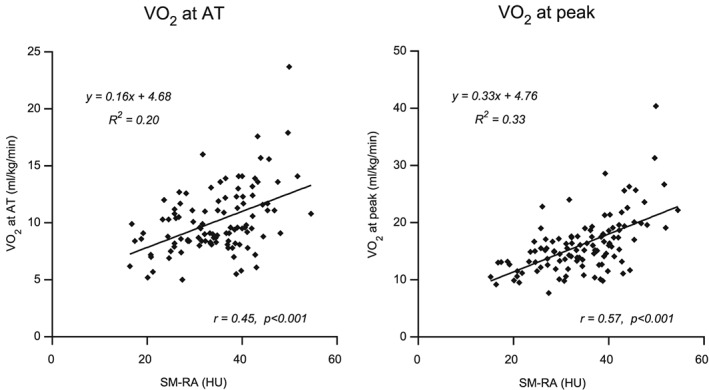
Correlations and regression plots of skeletal muscle radiation attenuation with $\dot{V}$O_2_ at AT (mL·kg^−1^·min^−1^) and $\dot{V}$O_2_ peak (mL·kg^−1^·min^−1^). Five patients did not reach their anaerobic threshold, and these patients were excluded from this analyses. HU, Hounsfield unit; SM‐RA, skeletal muscle radiation attenuation.

Univariate and multivariate linear regression results are shown in *Table* 3. Of the CT body composition parameters, SM‐RA had the strongest association with both weight‐adjusted $\dot{V}$O_2_ at AT and $\dot{V}$O_2_ peak in the univariate analysis (*R* ^2^ = 0.20 and 0.33, respectively; correlations, *r* = 0.45 and 0.57, respectively, *P* < 0.001; *Figure* 2). Skeletal muscle mass only showed a weak association with $\dot{V}$O_2_ peak (*R* ^2^ = 0.06; correlation, *r* = 0.24, *P* = 0.010). Other significant variables in the univariate analysis were age, BMI, ASA > 2, VAT, and SAT. However, in the multivariate analysis, only SM‐RA and age were added to the model because adding additional variables did not result in a significant *F* change (*P* > 0.05). In the multivariate analysis, SM‐RA was significantly associated with both weight‐adjusted $\dot{V}$O_2_ at AT (*B* = 0.12, 95% CI 0.06–0.18, *P* < 0.001, *R* ^2^ = 0.26) and $\dot{V}$O_2_ peak (*B* = 0.25, 95% CI 0.15–0.34, *P* < 0.001, *R* ^2^ = 0.42); see *Table* 3. Other body composition variables such as sarcopenia were excluded from the model after the addition of age.

**Table 3:** Univariate and multivariate linear regression analysis for $\dot{V}$O_2_ at anaerobic threshold (mL·kg^−1^·min^−1^) and $\dot{V}$O_2_ peak (mL·kg^−1^·min^−1^)

	$\dot{V}$O_2_ at AT (mL·kg^−1^·min^−1^)						$\dot{V}$O_2_ peak (mL·kg^−1^·min^−1^)					
	Univariate			Multivariate			Univariate			Multivariate		
	B	95% CI	P‐value	B	95% CI	P‐value	B	95% CI	P‐value	B	95% CI	P‐value
Age	−0.10	−0.14 to −0.58	<0.001*	−0.07	−0.11 to −0.02	0.004	−0.22	−0.28 to −0.15	<0.001*	−0.14	−0.21 to −0.07	<0.001*
Male	0.33	−0.77 to 1.43	0.549	—	—	—	1.95	0.16 to 3.73	0.033*	—	—	—
BMI	−0.10	−0.20 to −0.01	0.041*	—	—	—	−0.14	−0.30 to 0.03	0.115	—	—	—
ASA > 2	−1.17	−2.26 to −0.77	0.036*	—	—	—	−2.96	−4.74 to −1.19	0.001*	—	—	—
Pancreas	0.41	−0.64 to 1.46	0.442	—	—	—	0.14	−1.62 to 1.90	0.876	—	—	—
Malignant	0.49	−0.97 to 1.96	0.506	—	—	—	0.56	−1.83 to 2.94	0.645	—	—	—
mGPS				—	—	—				—	—	—
0 (ref)	—	—	—	—	—	—	—	—	—	—	—	—
1	−0.64	−2.22 to 0.95	0.428	—	—	—	−1.45	−4.14 to 1.25	0.289	—	—	—
2	−1.38	−2.80 to 0.04	0.056	—	—	—	−1.55	−3.92 to 0.82	0.197	—	—	—
SM	0.03	−0.02 to 0.08	0.252	—	—	—	0.11	0.03 to 0.19	0.010*	—	—	—
VAT	−0.02	−0.04 to 0.01	<0.001*	—	—	—	−0.04	−0.06 to −0.02	0.001*	—	—	—
SAT	−0.03	−0.04 to −0.01	0.001*	—	—	—	−0.05	−0.07 to −0.02	<0.001*	—	—	—
SM‐RA	0.16	0.10 to 0.22	<0.001*	0.12	0.06 to 0.18	<0.001*	0.33	0.24 to 0.42	<0.001*	0.25	0.15 to 0.34	<0.001*
VAT‐RA	0.06	−0.00 to 0.12	0.057	—	—	—	0.09	−0.01 to 0.20	0.071	—	—	—
SAT‐RA	0.02	−0.04 to 0.08	0.507	—	—	—	0.03	−0.06 to 0.13	0.496	—	—	—

Variables were added to multivariate analysis starting with the lowest *P*‐value in univariate analysis. Variables were only added to multivariate analysis if they significantly improved the model fit (*F* change: *P* < 0.05). For the multivariate models, *R* ^2^ = 0.20 [ $\dot{V}$O_2_ at AT (mL·kg^−1^·min^−1^)] and *R* ^2^ = 0.42 [ $\dot{V}$O_2_ peak (mL·kg^−1^·min^−1^)].

ASA, American Society of Anesthesiologists; BMI, body mass index; mGPS, modified Glasgow prognostic score; RA, radiation attenuation; SAT, subcutaneous adipose tissue; SM, skeletal muscle; VAT, visceral adipose tissue; WBC, white blood cell count.

* Significant *P*‐value < 0.05.

### Overall survival, mortality, and length of hospital stay

Low‐weight‐adjusted $\dot{V}$O_2_ peak was associated with increased 1 year mortality (21% vs. 8%, *P* = 0.045) and increased length of hospital stay (13.9 ± 11.0 vs. 10.6 ± 9.3 days, *P* = 0.048) in the univariate analysis (*Table* 1). One patient (<1%) died within 30 days, and three patients (2%) within 90 days (all with low SM‐RA). However, in the multivariate analysis, there was only a trend (odds ratio 2.99, 95% CI 0.92–9.74, p=0.070). In the multivariate Cox regression analysis, there was also a trend for low‐weight‐adjusted $\dot{V}$O_2_ peak and overall survival [hazard ratio (HR) 2.10, 95% CI 0.98–4.51, *P* = 0.056]. As there was an association between $\dot{V}$O_2_ peak and SM‐RA, we wanted to test whether a combination of low physical performance and myosteatosis would be a better predictor of overall survival and mortality. We therefore identified three patient phenotypes based on $\dot{V}$O_2_ peak and SM‐RA: (i) high $\dot{V}$O_2_ peak, (ii) low $\dot{V}$O_2_ peak only, and (iii) low $\dot{V}$O_2_ peak and myosteatosis (low SM‐RA). Indeed, patients with both low $\dot{V}$O_2_ peak and myosteatosis had significantly lower survival in the multivariate Cox regression analysis (HR 2.42, 95% CI 1.04–5.63, *P* = 0.040); see *Table* 4. There was no significant association with 1 year mortality. The only other variable associated with lower survival was an elevated mGPS (≥1) (HR 2.37, 95% CI 1.14–4.93, *P* = 0.021).

**Table 4:** Univariate and multivariate Cox regression and logistic regression analysis for overall survival and 1 year mortality

	Overall survival						1 year mortality					
	Univariate			Multivariate			Univariate			Multivariate		
	HR	95% CI	P‐value	HR	95% CI	P‐value	OR	95% CI	P‐value	OR	95% CI	P‐value
Age	1.02	0.98–1.05	0.341				1.01	0.97–1.06	0.673			
Male	0.66	0.30–1.45	0.303				0.81	0.28–2.34	0.700			
BMI	1.02	0.95–1.09	0.618				1.04	0.94–1.15	0.462			
ASA > 2	1.64	0.79–3.37	0.182				3.59	1.27–10.1	0.016*	3.08	0.99–9.54	0.051
Pancreas	1.62	0.78–3.37	0.201				2.28	0.92–8.27	0.071	3.29	0.97–11.2	0.057
Malignant	3.53	0.84–14.9	0.085	3.32	0.79–14.0	0.103	—	—	—†			
mGPS ≥ 1	2.39	1.17–4.88	0.017*	2.37	1.14–4.93	0.021*	2.41	0.87–6.70	0.090	1.84	0.58–5.84	0.304
Low SM	1.34	0.64–2.82	0.435				0.75	0.27–2.07	0.580			
Low SM‐RA	1.60	0.76–3.40	0.219				1.67	0.60–4.69	0.327			
Low $\dot{V}$O_2_ at AT	1.31	0.64–2.70	0.465				1.66	0.60–4.62	0.329			
Low $\dot{V}$O_2_ peak	2.04	0.95–4.36	0.066	2.10	0.98–4.51	0.056	2.97	0.99–8.93	0.052	2.99	0.92–9.74	0.070
Patient phenotype												
High $\dot{V}$O_2_ peak (reference)	—	—	—	—	—	—	—	—	—	—	—	—
Low $\dot{V}$O_2_ peak only	1.80	0.64–5.07	0.265	2.01	0.71–5.68	0.187	2.50	0.60–10.5	0.209	2.65	0.51–13.7	0.246
Low $\dot{V}$O_2_ peak and myosteatosis	2.31	0.99–5.35	0.050*	2.42	1.04–5.63	0.040*	3.10	0.95–10.2	0.061	3.21	0.90–11.5	0.073

Variables with *P* < 0.1 in univariate analysis were added to the multivariate analysis. Multivariate analysis for patient phenotypes was performed separately without including low SM, low SM‐RA, low $\dot{V}$O_2_ at AT, and low $\dot{V}$O_2_ peak variables.

ASA, American Society of Anesthesiologists; AT, anaerobic threshold; BMI, body mass index; mGPS, modified Glasgow prognostic score; RA, radiation attenuation; SM, skeletal muscle.

* Significant *P*‐value <0.05.

† Could not be included as the benign patients had 0% 1 year mortality.

## Discussion

In this study, myosteatosis (low SM‐RA) was associated with reduced fitness ( $\dot{V}$O_2_ at AT, $\dot{V}$O_2_ peak, and $\dot{V}$_E_/ $\dot{V}$CO_2_ slope) in both men and women, while sarcopenia (low skeletal muscle mass) was not. SM‐RA was associated with both weight‐adjusted $\dot{V}$O_2_ at AT and $\dot{V}$O_2_ peak in the multivariate analysis. Furthermore, combining low $\dot{V}$O_2_ peak with myosteatosis (low SM‐RA) was found to be a better predictor of overall survival than was low $\dot{V}$O_2_ peak or low SM‐RA alone. Additionally, an elevated mGPS was associated with lower overall survival. This novel finding demonstrates that CT‐derived SM‐RA data are associated not only with SM structure but also with SM functioning.

Sarcopenia in a cancer population is multifactorial, and while tumour burden may be one of the contributing factors, poor physical fitness is a major factor. In addition, several studies in HPB populations demonstrated that particularly myosteatosis is associated with poor overall survival and increased surgical complications.^24^, ^38^, ^39^ In the present study, the relationship between similar body composition and survival was not observed, as the present cohort was too heterogeneous for survival analysis. The approach of using a heterogeneous cohort provided a pragmatic snapshot capture of the HPB patient population that lends itself easily to external validation in other patient cohorts. In a series of 1473 consecutive patients with lung and abdominal cancer, the presence of low SM‐RA was a significant negative predictor. These data were corroborated in a study by Rollins *et al*.,^40^ where the prevalence of myosteatosis in patients with unresectable pancreaticobiliary cancers was found to be 55.3%. Furthermore, myosteatosis but not sarcopenia was significantly associated with a reduction in overall survival and systemic inflammation. In the present study, we were able to link myosteatosis with a high BMI and low fitness, but we only found a weak correlation between myosteatosis and both C‐reactive protein and albumin levels.

Although the relationship between myosteatosis, sarcopenia, and poor fitness was assumed, no study had ever set out to test this hypothesis. Interestingly, we observed that sarcopenia was only significantly associated with weight‐adjusted $\dot{V}$O_2_ peak in the univariate analysis but was excluded from the multivariate model after addition of age, suggesting that the association was age related rather than disease related. In addition, while sarcopenia can be a result of muscle loss that occurred over time, a single time point assessment of a patient's muscle mass was previously shown not to be associated with actual muscle loss over time.^30^ Indeed, skeletal muscle mass at a single time point is also affected by age, sex, race, build, and disease and hence might not fully correspond with muscle strength/function.^41^ Myosteatosis, on the other hand, was associated with reduced fitness ( $\dot{V}$O_2_ at AT, $\dot{V}$O_2_ peak, and $\dot{V}$_E_/ $\dot{V}$CO_2_ slope) in both multivariate analyses. Myosteatosis is generally regarded as the result of a pathologic process involving systemic inflammation and insulin resistance in disease states such as cancer cachexia or obesity.^23^ SM insulin resistance, redox dysfunction, and oxidative stress are associated with decreased glucose uptake and mitochondrial dysfunction, possibly leading to decreased muscle function and fitness.^42^, ^43^, ^44^

Lack of physical activity and fitness is a major modifiable risk factor of ill health^45^ and premature death. There is a large epidemiological body of evidence supporting the notion that physical fitness has benefits in almost every context of health and disease, advocating better outcomes for fitter people.^46^ Furthermore, physical *inactivity* is one of the leading public health issues we face,^47^, ^48^ and its association with cancer risk is quite clear.^49^ The biological bases underlying the associations between physical activity, fitness, and cancer risk are incompletely defined.^50^ The reliability and predictive value of perioperative objectively measured physical fitness using CPET in cancer patients are well established,^1^, ^51^, ^52^ with emerging evidence in pancreatic and hepatobiliary cohorts.^15^, ^18^, ^19^, ^53^ In this study, we found that $\dot{V}$O_2_ peak was the strongest predictor of adverse outcome (1 year mortality and length of hospital stay). Combining CPET with CT data could provide a better prediction of clinical outcome compared with either of them alone, as we found that patients with both low $\dot{V}$O_2_ peak and myosteatosis had significantly lower overall survival while low $\dot{V}$O_2_ peak only was not significantly associated with overall survival. Evidence has shown that a higher physical activity and fitness after a cancer diagnosis and its treatments reduce perioperative risk and improve quality of life and post‐operative outcomes including survival.^11^, ^13^, ^14^, ^50^, ^54^, ^55^

Such data raise the obvious hypothesis: Can health outcomes be improved by perioperative interventions targeted at improving body composition and physical fitness? Body composition and fitness modulation as a concept in surgical risk prediction is attractive due its potential reversibility. In elective surgical patients, there is a small window of opportunity from contemplation of surgery and tumour staging to the date of surgery. Tailored programmes during this period should be multi‐faceted and targeted at individual patients' needs. A combination of strategies targeting poor muscle function, reduced physical fitness, secondary anorexia, inflammation, psychological health, and poor nutrition has been suggested in the context of multimodal prehabilitation prior to cancer and major intra‐abdominal surgery.^56^, ^57^, ^58^, ^59^, ^60^, ^61^ A large body of evidence exists utilizing exercise to reverse loss of muscle mass and strength with ageing.^62^ In addition, physical exercise improves muscular strength and ameliorates systemic inflammation in cancer patients.^63^ Evidence relating to improving fitness with exercise in the perioperative period is also gaining momentum. While reduced length of stay, post‐operative morbidity, and critical care dependency have been observed in cardiothoracic^64^ patients undergoing prehabilitation programmes, there are limited data examining its subsequent impact on post‐operative outcomes following abdominal surgery.^59^, ^65^, ^66^ Moreover, understanding the underlying molecular mechanisms of muscle loss and interrogating distinct muscle phenotypes^67^ and their relationship to cancer surgery morbidity and survival are urgently needed. This is key to inform the development of interventions and treatment strategies to mitigate against poor outcomes.^35^

In conclusion, we found that myosteatosis and not sarcopenia (assessed by CT scan) was associated with physical fitness (assessed with CPET) in a surgical HPB population. These data suggest that a simple and fast analysis of myosteatosis on a single‐slice CT image provides information (albeit limited) on the patients' physical fitness. CT body composition combined with objectively measured fitness (CPET) might provide additive risk stratification benefits and guide personalized multimodal interventions during the perioperative period.

## Conflict of interest

Malcolm A. West was supported by a National Institute for Health Research Academic Clinical Lecturer Award (CL‐2016‐26‐002) and declares that he has no conflict of interest. David P. J. van Dijk was supported by the Netherlands Organisation for Scientific Research (NWO Grant 022.003.011) and declares that he has no conflict of interest. Fredrick Gleadowe declares that he has no conflict of interest. Thomas Reeves declares that he has no conflict of interest. John Primrose declares that he has no conflict of interest. Mohammed Abu Hilal declares that he has no conflict of interest. Mark R. Edwards declares that he has no conflict of interest. Sandy Jack declares that she has no conflict of interest. Sander S. S. Rensen declares that he has no conflict of interest. Michael P. W. Grocott declares that he has no conflict of interest. Denny Z. H. Levett declares that she has no conflict of interest. Steven W. M. Olde Damink declares that he has no conflict of interest.
